# A Deep Learning Framework for Vibration-Based Assessment of Delamination in Smart Composite Laminates

**DOI:** 10.3390/s20082335

**Published:** 2020-04-20

**Authors:** Asif Khan, Jae Kyoung Shin, Woo Cheol Lim, Na Yeon Kim, Heung Soo Kim

**Affiliations:** 1 Department of Mechanical, Robotics and Energy Engineering, Dongguk University-Seoul, 30 Pil-dong 1 Gil, Jung-gu, Seoul 04620, Korea; khanuet11@gmail.com (A.K.); jkshin@onepredict.com (J.K.S.); luc2978@dgu.edu (W.C.L.); nayeunk@naver.com (N.Y.K.); 2 OnePredict Co., Ltd Solution I R&D Division, Seoul 08826, Korea

**Keywords:** delamination, smart composite laminates, structural vibration, spectrograms, deep learning

## Abstract

Delamination is one of the detrimental defects in laminated composite materials that often arose due to manufacturing defects or in-service loadings (e.g., low/high velocity impacts). Most of the contemporary research efforts are dedicated to high-frequency guided wave and mode shape-based methods for the assessment (i.e., detection, quantification, localization) of delamination. This paper presents a deep learning framework for structural vibration-based assessment of delamination in smart composite laminates. A number of small-sized (4.5% of total area) inner and edge delaminations are simulated using an electromechanically coupled model of the piezo-bonded laminated composite. Healthy and delaminated structures are stimulated with random loads and the corresponding transient responses are transformed into spectrograms using optimal values of window size, overlapping rate, window type, and fast Fourier transform (FFT) resolution. A convolutional neural network (CNN) is designed to automatically extract discriminative features from the vibration-based spectrograms and use those to distinguish the intact and delaminated cases of the smart composite laminate. The proposed architecture of the convolutional neural network showed a training accuracy of 99.9%, validation accuracy of 97.1%, and test accuracy of 94.5% on an unseen data set. The testing confusion chart of the pre-trained convolutional neural network revealed interesting results regarding the severity and detectability for the in-plane and through the thickness scenarios of delamination.

## 1. Introduction

Composite materials are continuously replacing conventional metallic materials in a variety of industries (e.g., aerospace, automotive) due to their lightweight, high specific strength, high specific stiffness, and design flexibility. Besides, composite materials do not present the typical corrosion problems of conventional metallic materials [1,2,3,4]. However, owing to their anisotropic characteristics, laminated composite materials suffer from a variety of complex manufacturing defects and in-service damages such as air trapped between plies, non-uniform distribution of epoxy, inadequate consolidation pressure, delamination, matrix cracking, and fiber fracture, among others [5,6,7,8]. The presence of defects in laminated composites is more critical in terms of effects and more challenging in terms of assessment (i.e., detection, quantification, and localization) than those in the metallic materials. The presence of delamination or separation between plies is one of the most detrimental defects in the laminated composite as it can cause up to 60% loss in the strength without any perceivable effects at the surface or noticeable change in the stiffness [9,10]. Once initiated, delamination could propagate into a wide damage zone and thus compromising the structural integrity of the component or structure [11,12]. To ensure safe and reliable operation of laminated composite materials in real engineering structures, it is imperative to timely identify, quantify, and localize the presence of delamination in these materials.

The anisotropic material properties and complex microstructure make the damage assessment of composite materials more challenging than metallic materials. Consequently, the damage assessment of laminated composite is an active research area where continuous efforts are made to find cost-effective techniques for the detection, quantification, and localization of delamination in these materials [13,14,15,16]. Various non-destructive testing (NDT) procedures are being used for the assessment of delamination such as acoustic emission, thermography, ultrasonic, and X-ray [17,18,19,20,21]. However, most of the NDT techniques are often costly, labor-intensive, unable to handle large size test objects, and depend heavily on the expertise of the operator. Hence, continuous research efforts are underway to come up with new techniques for the damage assessment of laminated composites. Zhao et al. [22] proposed a local wavenumber method for the localization and characterization of mode 1 delamination in carbon fiber/epoxy composite laminates. The wave propagation velocity was found to transform at the delamination and an increase in the central frequency was found to cause larger attenuation and dispersion in the guided waves. Grassia et al. [23] developed a strain-based method for structural health monitoring of composite structures by making a fingerprint model of the reference healthy structure via a neural network. The developed method was able to detect damages from the early stage of micro-cracks up to delamination in composite structures. Yang et al. [24] proposed that the variations of modal frequency under mass loading could be used to quantify the effects of delamination in laminated composites. Mei et al. [25] studied high-frequency local vibration for detecting and quantifying the size, shape, and depth of delamination in composite plates. Sikdar et al. [26] investigated the effects of debonding and variable ambient temperature on the propagation of Lamb wave in composite materials. The presence of delamination was reflected by a reduction in the amplitudes and velocity of A_0_ mode of the Lamb wave. The increase in temperature further decreased the propagation velocity and amplitudes of A_0_ mode. Temperature correction factors were proposed to minimize the effect variable temperature conditions on structural health monitoring strategies for composite materials. Kundu et al. [27] proposed a generic machine learning framework for the detection and localization of damage in composite materials using spatially and temporally correlated features of an acoustic emission signal. Khan et al. [28] presented a machine learning paradigm for the detection, quantification, and prediction of delamination in smart composite laminates. Pre-trained predictive models were found to predict physically reasonable labels for new unseen cases of delamination. Chen et al. [29] used a laser Doppler vibrometer and curvatures of the operating deflection shapes (CODS) for the detection and location of delamination in laminated composites. It was found that third and fifth pure bending modes show more sensitivity to the presence of local delaminations. Yelve et al. [30] proposed a Lamb wave-based nonlinear method for the assessment of delamination in laminated composites. A spectral damage index was extracted from the fundamental and higher harmonics for assessing the size of delamination, while spectral and temporal data were used for the localization of delamination. Feng et al. [31] studied the propagation and dispersion of Lamb waves in delaminated composite laminates. It was found that the delamination length could be quantified from the phase difference of two waves travelling in the upper and lower sub-laminates. Dafydd et al. [32] investigated the potential of ultrasonic guided waves for the severity assessment of impact damage in carbon-fiber-reinforced polymers. Among the two fundamental modes of *A_0_* and *S_0_*, *A_0_* mode showed more sensitivity to the damage severity than *S_0_*. Li et al. [33] developed a numerical model for the propagation and dispersion of ultrasonic guided waves in damaged composite using high-fidelity local interaction simulation. The time-frequency features obtained via the matching pursuit decomposition algorithm showed that a signal from symmetric excitation was more sensitive to damage location than the signal from the anti-symmetric excitation. Huang et al. [34] studied principal component analysis (PCA) for the detection and quantification of delamination in smart composite laminates. It was found that distance between the healthy and delaminated data clusters could be used to quantify the severity of delamination. 

In general, the vibratory responses in the fundamental modes (e.g., structural vibration) carry information about the presence of damage (i.e., global behavior) in the structure, whereas information on the location of damage is captured by high-frequency Lamb waves and acoustic emission [35]. In the published literature on damages in laminated composites, the presence of delamination had been assessed from the structural vibration and modal parameters [36], whereas guided waves and acoustic emission had been used for the detection and localization of delamination [37,38]. However, some practical limitations of the methods that employ high-frequency waves for nondestructive evaluation are their need of too many sensors, optimized location of the receivers, the damage to be between the actuator(s) and sensor(s), data acquisition at a higher rate, and complex signal processing [39,40,41]. On the contrary, structural vibration is more readily available for the assessment of damage due to its easy measurement via smart materials [42]. In addition, the delamination is not necessary to be in the path between the sensor and actuator, unlike the guided wave-based methods. Hence, in this work, an effort has been made to use low-frequency structural vibration for the local and global assessment of inner and edge delaminations in laminated composites. 

From the contemporary research on delamination in laminated composites, it can be found that most of the research efforts are dedicated to the use of higher-frequency guided waves (e.g., Lamb waves), acoustic emission/acoustic ultrasonic, and mode shape curvatures for the detection, quantification, and localization of delamination [43,44,45,46,47,48,49]. This paper proposes a deep learning framework for the assessment of delamination in piezo-bonded laminated composites using low-frequency structural vibration responses. An electromechanically coupled mathematical model of the laminated composite with piezoelectric sensors and actuators is developed and solved in the time domain. Several cases of inner and edge delaminations are simulated in a smart cantilever plate. The transient responses of the healthy and delaminated smart composite laminates are transformed into spectrograms, which are processed with the deep convolutional neural network (CNN). Contrary to conventional hand-crafted discriminative features, the CNN automatically extracts discriminative features from the spectrogram and uses those features to classify the healthy and delaminated smart composite laminates. The obtained results showed that the proposed approach could be employed for the assessment of inner and edge delaminations in smart composite laminates while using low-frequency structural vibration only. 

## 2. Problem Formulation

The accurate description of the structural deformation of a laminated composite with surface bonded or embedded piezoelectric sensors and actuators requires a coupled electromechanical formulation. The kinematics of the smart structure with single and multiple discrete delaminations is developed based on improved layerwise theory [50], whereas higher-order electric potential function [51,52] is used to model the potential variation through the piezoelectric patches. As per the improved layerwise theory, first-order shear deformation theory is superimposed with layerwise functions to describe the displacements of a point with coordinates (*x*, *y*, *z*) as follows [34]:

(1) $$\begin{array}{ll} U_{i}^{k}\left(x,y,z,t\right)= & u_{i}\left(x,y,t\right)+A_{i}^{k}\psi_{1}+B_{i}^{k}\psi_{2}+C_{i}^{k}w_{,x}+D_{i}^{k}w_{,y}\\ & +E_{i}^{k}\left\{{\bar{w}}_{,x}^{j}\right\}+F_{i}^{k}\left\{{\bar{w}}_{,y}^{j}\right\}+\sum_{j=1}^{N-1}{\bar{u}}_{i}^{j}H\left(z-z_{j}\right)\\ U_{3}^{k}\left(x,y,z,t\right)= & w\left(x,y,t\right)+\sum_{j=1}^{N-1}{\bar{w}}^{j}H\left(z-z_{j}\right) \end{array},$$

where $U_{i}^{k}\left(i=1,2\right)$ and $U_{3}^{k}$ refer to the in-plane displacements and transverse deflection, respectively. The terms $u_{i}\left(i=1,2\right)$ and w respectively denote the in-plane and transverse displacements at the mid-plane of the laminate. The symbol $\psi_{i}\left(i=1,2\right)$ describes the rotation of normal to the mid-plane, and thus accounts for the shear stress variation through the thickness of the laminate. The terms of ${\bar{u}}_{i}^{j}$ and ${\bar{w}}^{j}$ account for the possible slipping and jump in the displacements at the debonded interfaces between adjacent plies, H denotes Heaviside function, and $z_{j}$ refers to the delaminated interface. The terms $A_{i}^{k},B_{i}^{k},C_{i}^{k},D_{i}^{k},E_{i}^{k}$, and $F_{i}^{k}$ are layerwise coefficients which are calculated in terms of the material and geometric properties of the laminates [50]. In the displacement field of Equation (1), perfectly bonded and delaminated interfaces are modeled by setting the terms ${\bar{u}}_{i}^{j}$ and ${\bar{w}}^{j}$ to zero and nonzero, respectively.

The electric potential field of the piezoelectric sensors and actuators is assumed to have a cubic distribution along the thickness direction. The mathematical description of the potential field for the *p*-th piezoelectric layer is given by Equation (2) [51]:

(2) $$\begin{array}{ll} \phi^{p}\left(x,y,z,t\right) & =\phi_{0}^{p}\left(x,y,t\right)-\left(z-z_{0}^{p}\right)E_{z}^{p}\left(x,y,t\right)+4\left(\frac{z-z_{0}^{p}}{h^{p}}\right)\\ & \times \left[\left(z-z_{0}^{p}\right)\left(\frac{{\bar{\phi }}^{p}\left(x,y,t\right)}{h^{p}}+E_{z}^{p}\left(x,y,t\right)\right)-\phi_{0}^{p}\left(x,y,t\right)\right] \end{array},$$

where $\phi_{0}^{p}$ and $E_{z}^{p}$ refer to the electric potential and electric field at the mid-plane of the *p*-th piezoelectric layers, respectively. The symbol ${\bar{\phi }}^{p}$ shows the potential difference between the top and bottom electrodes, $z_{0}^{p}$ and $h^{p}$ refer to the mid-plane position and thickness of the piezoelectric patch, respectively. The 2nd and 3rd terms of Equation (2) respectively denote the linear and nonlinear potential variation through the thickness of the piezoelectric layer.

In the current work, linear constitutive relations (i.e., constant material coefficients) are considered for the converse and direct piezoelectric effects. The constitutive relations of piezoelectric actuator and sensor are shown by Equation (3) [51]:

(3) $$\begin{array}{l} \sigma_{ij}=c_{ijkl}\varepsilon_{kl}-e_{ijk}E_{k}\\ D_{i}=e_{ijk}\varepsilon_{jk}+b_{ij}E_{j} \end{array},$$

where $\sigma_{ij}$ and $D_{i}$ denote the stress tensor and electric displacement vector, respectively. The quantity $\varepsilon_{ij}$ and $E_{i}$ respectively denote the strain tensor and the electric field vector. The terms $c_{ijkl}$ and $e_{ijk}$ refer to the elastic and piezoelectric constants, respectively. The term $b_{ij}$ denotes the dielectric permittivity of the piezoelectric material. For the linear piezoelectricity of Equation (3), the electric field vector $E_{i}$ is obtained from the scalar potential field of Equation (2) as follows:

(4) $$E_{i}=-\phi_{,i}\;(i=1,2,3),$$

where the subscript denotes partial derivative with respect to *i* with *i* = 1, 2, 3. 

A finite element method is adopted to combine the displacement and potential fields for a 4-noded plate element. The primary in-plane unknowns ($u_{1},u_{2},\psi_{1},\psi_{2},{\bar{u}}_{1}^{j},{\bar{u}}_{2}^{j}$) of the displacement field and electrical unknowns ($\phi_{0}^{p},E_{z}^{p}$) of the potential field are described in terms of linear Lagrange interpolation functions, whereas Hermite cubic interpolation functions are used for out-of-plane unknowns of the displacement field, as shown by Equation (5) [51]:

(5) $$\begin{array}{l} \left(u_{1},u_{2},\psi_{1},\psi_{2},{\bar{u}}_{1}^{j},{\bar{u}}_{2}^{j}\right)=\sum_{m=1}^{4}N_{m}\left[{\left(u_{1}\right)}_{m},{\left(u_{2}\right)}_{m},{\left(\psi_{1}\right)}_{m},{\left(\psi_{2}\right)}_{m},{\left({\bar{u}}_{1}^{j}\right)}_{m},{\left({\bar{u}}_{2}^{j}\right)}_{m}\right]\\ \left(\phi_{0}^{p},E_{z}^{p}\right)=\sum_{m=1}^{4}\left[N_{m}{\left(\phi_{0}^{p}\right)}_{m},N_{m}{\left(E_{z}^{p}\right)}_{m}\right]\\ w=\sum_{m=1}^{n}\left[H_{m}{\left(w\right)}_{m}+H_{xm}\left(w_{,x}\right)+H_{xm}{\left(w_{,y}\right)}_{m}\right]\\ {\bar{w}}^{j}=\sum_{m=1}^{n}\left[H_{m}{\left(\bar{w}\right)}_{m}+H_{xm}\left({\bar{w}}_{,x}\right)+H_{xm}{\left({\bar{w}}_{,y}\right)}_{m}\right] \end{array},$$

where $N_{m}$ refers to Lagrange interpolation function and $H_{m},H_{xm},H_{ym}$ are Hermite interpolation functions.

From Equation (5), the displacement unknowns can be described in terms of nodal unknowns by using the matrix notation as follows:

(6) $$\begin{array}{l} \left\{u_{u}^{e}\right\}=\left[N_{u}\right]\left\{d_{u}\right\}\\ \left\{u_{\phi }^{e}\right\}=\left[N_{\phi }\right]\left\{d_{\phi }\right\} \end{array},$$

where

(7) $$\begin{array}{l} \left\{u_{u}^{e}\right\}={\left[u_{1},u_{2},w,\psi_{1},\psi_{2},{\bar{u}}_{1}^{j},{\bar{u}}_{2}^{j},{\bar{w}}^{j}\right]}^{T}\\ \left\{d_{u}\right\}=\left[\cdots ,u_{1i},u_{2i},w_{i},w_{,xi},w_{,yi},\psi_{1i},\psi_{2i},{\bar{u}}_{1i}^{j},{\bar{u}}_{2i}^{j},{\bar{w}}_{,i}^{j}{\bar{w}}_{,xi}^{j},{\bar{w}}_{,yi}^{j},\cdots \right]\\ \left\{u_{\phi }^{e}\right\}={\left[\phi_{0}^{p},E_{z}^{p}\right]}^{T}\\ \left\{d_{\phi }\right\}={\left[\cdots ,\phi_{0i}^{p},E_{zi}^{p},\cdots \right]}^{T} \end{array}.$$

In terms of the displacement field (Equation (1)) and electric potential field (Equation (2)), the elemental displacement field u(x,y,z,t), elemental strain field ε(x,y,z,t), and electric potential field $E^{p}\left(x,y,z,t\right)$ can be described as follows:

(8) $$\begin{array}{l} u\left(x,y,z,t\right)=L_{u}u_{u}^{e}\left(x,y,t\right)\\ \varepsilon \left(x,y,z,t\right)=L_{\varepsilon }u_{u}^{e}\left(x,y,t\right)\\ \phi^{p}\left(x,y,z,t\right)=V_{b}\left(z-z_{0}^{p},h^{p},{\bar{\phi }}^{p}\right)+L_{\phi }^{p}u_{\phi }^{p}\left(x,y,z,t\right)\\ E^{p}\left(x,y,z,t\right)=-F_{b}\left(z-z_{0}^{p},h^{p},{\bar{\phi }}^{p}\right)-L_{E}^{p}u_{\phi }^{j}\left(x,y,z,t\right) \end{array},$$

where the operators $L_{u},L_{\varepsilon },L_{\phi }^{p},L_{E}^{p}$ and the expressions for $V_{b}$ and $F_{b}$ are shown in Appendix A. The equation of motion is obtained by using the variational principle as shown by Equation (9):

(9) $$\begin{array}{l} \delta \pi_{u}=\int_{0}^{t}\left[\int_{V}\left(\rho {\ddot{u}}_{i}\delta u_{i}+\sigma_{ij}\delta \varepsilon_{ij}+\xi {\dot{u}}_{i}\delta u_{i}\right)dV+\int_{S}t_{i}\delta u_{i}dS\right]dt=0\\ \delta \pi_{\phi }=\int_{0}^{t}\left[\int_{V}D_{i}\delta \phi_{,i}dV+\int_{S}q_{e}\delta \phi dS\right]dt=0 \end{array},$$

where $\delta \pi_{u}$ and $\delta \pi_{\phi }$ refer to the energy functional of the mechanical and electrical fields, respectively. The terms ρ denotes mass density, ξ is damping ratio, $t_{i}$ is traction vector, and $q_{e}$ represents charge density.

The electromechanically coupled equation of motion is obtained by substituting the stress, strain, and electric displacement components in Equation (9), and is shown in matrix form by Equation (10) [51]:

(10) $$\begin{array}{r} M{\ddot{d}}_{u}+C{\dot{d}}_{u}+K_{uu}d_{u}+K_{u\phi }d_{\phi }=F_{u}\\ K_{\phi u}d_{u}+K_{\phi \phi }d_{\phi }=F_{\phi } \end{array},$$

where M and C respectively denote structural mass and damping matrices. The terms $K_{uu}$ and $K_{\phi \phi }$ refer to the stiffness matrices of the mechanical and electrical fields, respectively. The symbols of $K_{u\phi }$ and $K_{\phi u}$ show the electromechanical coupling matrices, the vectors $d_{u}$ and $d_{\phi }$ refer to the displacement unknowns and electrical unknowns of piezoelectric patches. The terms $F_{u}$ and $F_{\phi }$ denote the applied mechanical and electrical forces, respectively. The actuation and sensing mechanism of the piezoelectric actuator and sensors is accounted for by the piezoelectric–mechanical coupling matrices of $K_{u\phi }$(converse piezoelectric effect) and $K_{\phi u}$(direct piezoelectric effect). The coupling matrices allow the piezoelectric actuator to produce mechanical actuation forces under input voltages and the piezoelectric sensors to generate electrical signals under mechanical deformation. 

For numerical solution, the mathematical model of Equation (10) is modified to Equation (11) by applying matrix condensation:

(11) $$M{\ddot{d}}_{u}+C{\dot{d}}_{u}+\left(K_{uu}-K_{u\phi }K_{\phi \phi }^{-1}K_{\phi u}\right)d_{u}=F_{u}-K_{u\phi }K_{\phi \phi }^{-1}F_{\phi }.$$

The developed numerical model was implemented in MATLAB. Newmark’s time integration algorithm [34] was employed to solve the electromechanically coupled model of Equation (11) in the time domain. In the algorithm, Taylor’s expansions with terms up to the second derivative were used to approximate the displacement variable, its first and second derivatives. The governing equation was reduced to a set of algebraic equations that were solved through an iterative process involving the force input at each time step and the initial conditions. 

## 3. Numerical Example

**Figure 1:**
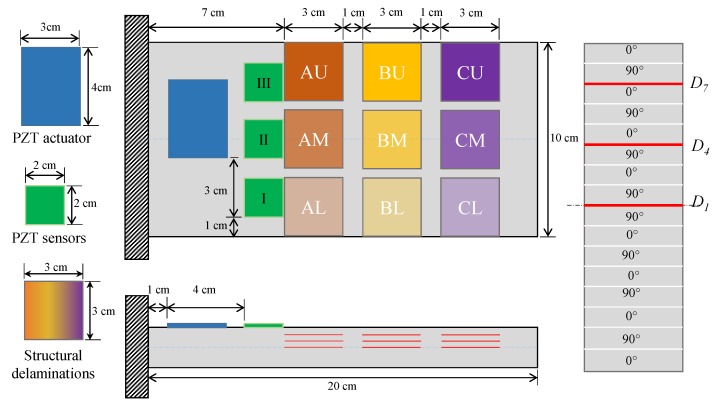
Schematic of smart composite laminate with various inner and edge delaminations (**left**) Top and front views (**right**) exaggerated view of the thickness direction.

The mathematical formulation of Section 2 is numerically implemented on a smart laminated composite plate with various cases of small sized (4.5% of the total area) inner and edge delaminations. The smart plate is made of 16-layers which are stacked together in a symmetric cross-ply configuration ([0/90]_4s_). One piezoelectric actuator and three piezoelectric sensors are attached to the surface of the smart plate as shown in the schematic of Figure 1.

Herein, the piezoelectric actuator is attached at the off-center position along the width direction so that mix-vibrating modes (i.e., bending and twisting) of the smart plate could be stimulated. The piezoelectric actuator and sensors are made of the same materials. Table 1 and Table 2 respectively show the material properties of a single lamina of the laminated composite and piezoelectric material.

In Figure 1, the symbols *AM*, *BM*, and *CM* denote the inner delaminations, whereas *AL*, *AU*, *BL*, *BU*, *CL*, and *CU* refer to the cases of edge delaminations. The inner and edge delaminations could occur at three different interfaces of *D_1_* (mid-plane), *D_4_*, and *D_7_* through-the-thickness of the smart plate. The in-plane delaminations combined with through-the-thickness interface are named by adding the respective interface number with the name of the in-plane location. For example, *AL1* means the presence of delamination at the *AL* in-plane location and at the *D_1_* interface. The smart plate without any defect is referred to as the ‘*Healthy* (*H*)’ case. The nine in-plane delaminations combined with three options of through-the-thickness interfaces add up to 27 cases of delaminations in the smart plate. 

**Figure 2:**
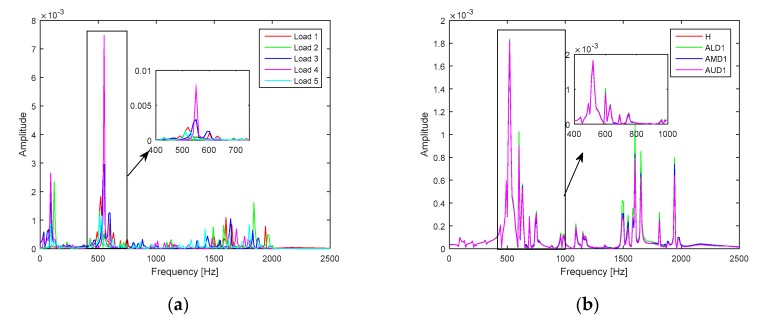
Frequency spectrum of the transient response for: (**a**) a single case (*AL1*) to 5 random loadings; (**b**) Healthy and different delaminated cases to a single random load.

In the FEM model of the smart plate, the host laminate and the piezoelectric patches were discretized with four-node plate elements. The healthy and delaminated smart plates were excited into random low-frequency vibration through a set of 1000 random harmonic voltages applied through the piezoelectric actuator and the corresponding responses were measured through the piezoelectric sensors. Figure 2a shows the response spectrum of *AL1* to the 5 random loadings as measured by sensor I, whereas Figure 2b shows the frequency response spectrum of *H*, *AL1*, *AM1*, and *AU1* to one of the random inputs as measured by sensor I.

The frequency spectrum of the transient responses in Figure 2a shows that random loadings stimulated multiple modes of vibration and caused more variability in the transient response of a single case (*AL1*). On the other hand, the variations caused by different damage cases (Figure 2b) is very subtle for different cases of in-plane delaminations and would further decrease for different interfaces of delaminations. The comparison of Figure 2 shows that conventional time and frequency domain discriminative features (e.g., mean, kurtosis, variance, skewness, etc.) could not be employed to discriminate the healthy plate from the delaminated plate. 

In general, the variations of modal parameters (i.e., natural frequency, damping, vibration mode shapes) are commonly used for the detection of delamination in the interlaminar regions of composite materials. However, model parameters have been mostly used for the detection of delamination and limited research effort can be found on the use of variation in modal parameters for the detection, quantification, and localization of delamination. Besides, monitoring variations in the modal damping and measurement of vibration mode shapes for delamination detection are challenging tasks, especially in practical experiments. In addition, the variations of the natural frequencies usually reflect the effect of large size delamination, and smaller delamination may not show any noticeable variation in the natural frequencies. Although, from Figure 2b, certain cases of delamination may show some difference of amplitude in comparison with the healthy case, the same trend could not be observed for all cases of delamination due to difference in the severity levels of different delamination. The less severe cases of delamination (e.g., AMD7, BMD7, CMD7, etc.) would not show any appreciable difference for the healthy and delaminated smart composite laminates. In addition, the variations in Figure 2b are smaller than in Figure 2a and the changes in natural frequency could not be used for the assessment of inner and edge delaminations in the laminated plate.

## 4. Proposed Methodology

**Figure 3:**
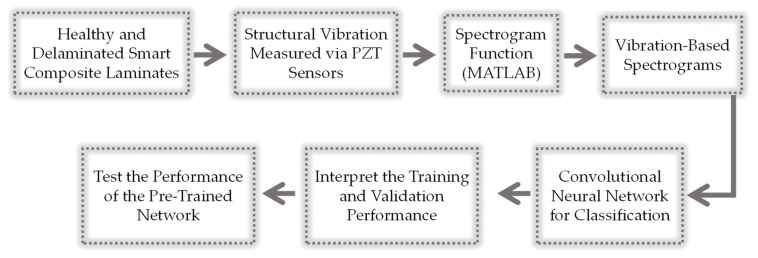
Schematic of the deep learning-based methodology for structural vibration-based delamination assessment of smart composite laminates.

The frequency spectrums in Figure 2 show that the structural vibration responses from the healthy and delaminated smart composite plates are more influenced by operating conditions than the delaminations in the plate. The randomness of loadings hinders the use of statistical time domain and frequency domain features for distinguishing the healthy case from the delaminated cases as well as a distinction between different cases of delaminations (e.g., *AL1*, *AL2*, *AL3*, *AM1*, etc.). Deep learning offers a natural solution to the current problem due to its capability to automatically extract discriminative features from the input images [53]. Figure 3 shows a schematic of the deep learning-based methodology for the current problem. 

**Figure 4:**
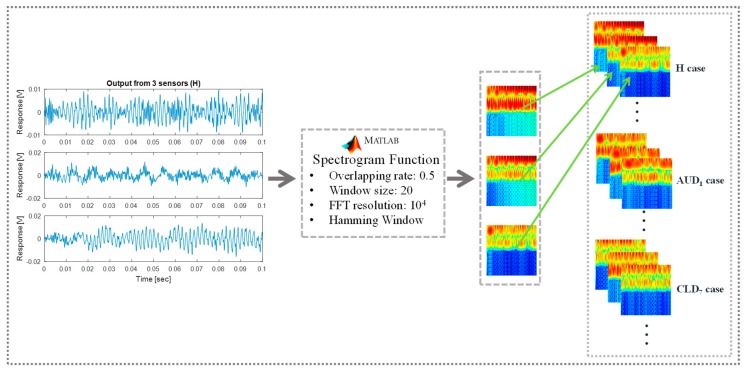
Preparation of vibration-based spectrograms for deep learning using the spectrogram function of Matlab.

Herein, the response from the three piezoelectric sensors is transformed into time-frequency spectral images using the *spectrogram* function in Matlab. The spectrogram function allows for controlling the time and frequency resolution of the spectrograms by adjusting the variables of *window size*, type of window function (*Hamming*, *Hann*, and *Black* window function), fast Fourier transform *(FFT) resolution*, and the *overlapping rate*. In general, the FFT resolution was observed to affect the spectral resolution of the vibration spectrograms and the computational time of the short time Fourier transform. In the current work, the FFT resolutions of 10, 10^2^, 10^4^, and 10^8^ were considered for initial assessment and the spectrogram with FFT resolution of 10^4^ and 10^8^ showed good spectral resolution. Although no significant difference was observed in the resolution of spectrograms with 10^4^ and 10^8^ FFT resolutions, the computational time for 10^8^ FFT resolution was about 3 times longer than for 10^4^ FFT resolution. Hence, an FFT resolution of 10^4^ was selected. The Hamming window function was selected due to its better performance in the high-frequency band compared with Hann and Blackman window functions. To prevent Picket fence error and avoid loss of information at the beginning and end of the signals [54], a 50% overlapping range was selected for the window function. In order to check the size of the window, which is the remaining resolution parameter, the spectral images were generated by specifying the size of the window as 10, 20, and 50. It was observed that smaller window size resulted in high time resolution and low frequency resolution, and vice versa. Hence, the size of the window was set to 20 to obtain a spectral image with an appropriate balance of time and frequency resolution. For the convolutional neural network (CNN), the spectrograms of the three sensors for a single loading were staked and supplied as nine-channel input (3 RGB channels for each sensor) as shown in Figure 4.

**Figure 5:**
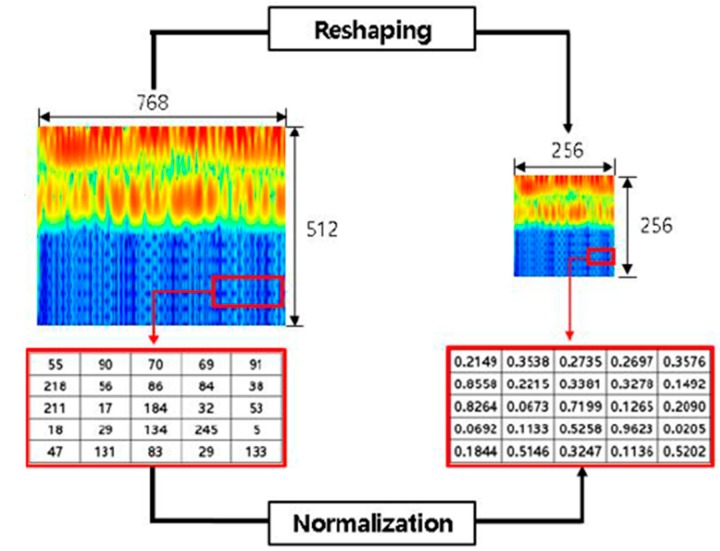
Size conversion and normalization of spectral images.

The original size of each spectrogram was 768 × 512 × 3 (width × height × number of channels) with the pixel values between 0 and 255. To reduce the computation cost of the deep learning algorithm and remove the divergence from the pixel values, each one of the three spectrograms was reduced to 256 × 256 × 3 (width × height × number of channels) and normalized to a pixel value between 0 and 1 as shown by Figure 5.

The goal of normalization was to change the pixel values of the spectrograms to a common scale for the healthy and delaminated structures without distorting the differences in the range of values. In addition, normalization reduced the influence of larger variance in the pixel values on the classification results of the deep learning algorithm. Details of the CNN-based classifier, interpretation of the training and validation process, and testing of the pre-trained network are discussed in the next section.

## 5. Results and Discussion

**Figure 6:**
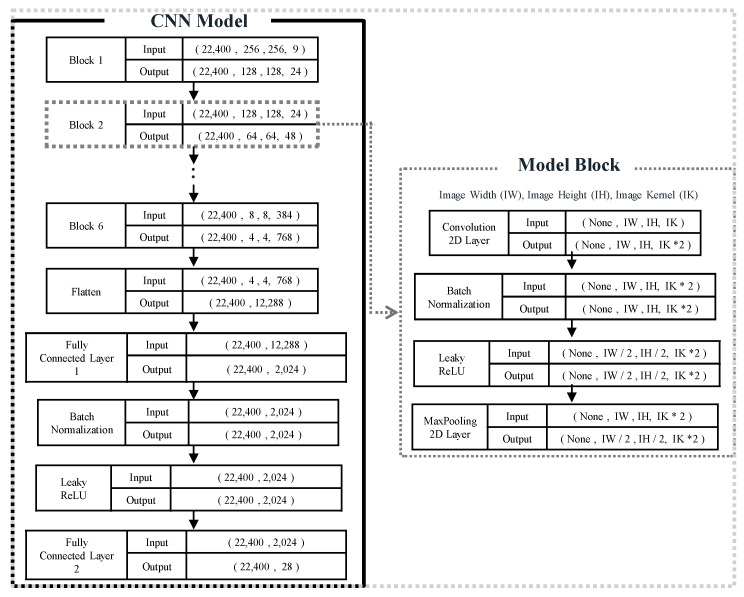
Architecture of the convolutional neural network for the delamination assessment in smart composite laminates.

The data set of vibration-based spectrograms consisted of one healthy case (*H*) and 27 cases of inner and edge delaminations in the smart laminated composite plate. Based on the applied random harmonic loads, each one of the 28 cases is comprised of 1000 instances. From the discussion in Section 4, each instance consists of 3 RGB spectrograms for one random load. The 28,000 spectrograms were split into 22,400 (80%) spectrograms for training, 2800 (10%) for validation of the network during training, and 2800 (10%) were used for evaluating the testing performance of the pre-trained network. A block diagram of the convolutional neural network used for the classification of vibration-based spectrograms is shown in Figure 6. 

Herein, the input to the network consists of 22,400 training spectrograms where the size of each spectrogram is equal to 256 × 256 × 9 (width × height × number of channels). In the network, six model blocks were used to automatically extract discriminative features from the spectrograms. Each model block was comprised of a 2D convolution layer with a filter/kernel of size 3 × 3, batch normalization, Leaky ReLU [55,56] as a nonlinear activation function, and a 2D max-pooling with a filter of size 2 × 2. Batch normalization and 2D max-pooling layers were used in each model block to prevent the network from overfitting of the training data. The kernel size of 3 × 3 was kept constant for all the 2D convolutional layers of the six model blocks. Whereas, the number of kernels in the six model blocks was respectively chosen to be 24, 24 × 2, 24 × 4, 24 × 8, 24 × 16, and 24 × 32. The three-dimensional output (4, 4, 768) from the last model block is converted into one-dimensional data (12,288) using a Flatten layer. After that, the data is passed through a fully connected hidden layer with 2024 neurons, batch normalization, the activation function of Leaky ReLU. The features are finally passed to a fully connected output layer with 28 neurons. The mathematical details and functionality of each layer of the CNN can be referred to in the references [57,58]. In the last fully connected layer of the network, a softmax function with a built-in function of TensorFlow (i.e., softmax_cross_entropy_with_logits_v2) was used to classify the 28 scenarios of the healthy and delaminated smart composite laminates. The learnable parameters of weights and bias were randomly initialized and trained from scratch. The hyperparameters of learning rate, number of hidden layers, and size and number of convolutional kernels were decided through an empirical experiment for the given dataset of healthy and delaminated smart composite laminates. The proposed CNN was trained up to 200 epochs with a learning rate of 0.0001 and Adam optimizer was used for fast calculation. CNN architecture was implemented using the open-source platform of TensorFlow on a GeForce RTX 2080 Ti GPU from NVIDIA.

The proposed architecture of the CNN was trained through a 10-fold cross-validation technique. The network showed an overall all training accuracy of 99.9% and validation accuracy of 97.1%. The high validation accuracy showed that the network had not overfitted the training data.

**Figure 7:**
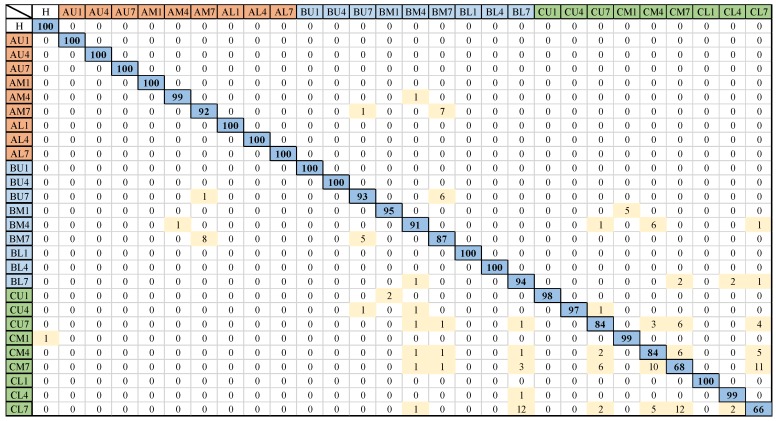
Confusion matrix of the pre-trained convolutional neural network (CNN) on unseen test data.

To evaluate the performance of the trained network, the pre-trained network was tested with 2800 spectrograms that were not used during the training and validation process. The pre-trained network showed a predictive test accuracy of 94.5% and Figure 7 depicts per-class predictive performance in the form of a confusion chart.

Herein, the labels in the first column denote the ground truth or actual classes, the topmost row shows the labels predicted by the pre-trained convolutional neural network, the numbers on the main diagonal represent the percentage of correctly classified instances, and the off-diagonal shows the percentage of incorrectly classified instances. It is worth noting that delamination at the mid-plane interface shows more noticeable effects in terms of stiffness change and dynamic response characteristics than delamination of the same size near the free surfaces (away from mid-plane) [50,59]. In addition, due to the high strain near the fixed end of the cantilever plate and proximity of the piezoelectric sensors, delamination near the fixed end would more pronouncedly affect the dynamic response than delamination of the same size near the free end of the smart plate. The test confusion matrix of Figure 7 reveals the following results:

1. The classifier has distinguished the healthy case from the delaminated cases with 100% accuracy.
2. The more severe cases of delaminations (the one that occurs at the mid-plane interface) have been identified with 95%~100% accuracy.
3. The pre-trained network can distinguish the inner delamination (*AM*, *BM*, and *CM*) from the edge delaminations (*AL*, *AU*, *BL*, *BU*, *CL*, and *CU*) with 90%~100% accuracy.
4. The major loss of accuracy is due confusion between the least severe cases of delaminations (i.e., inner and edge delaminations that occur near the free surface and whose position is furthest from the sensors). More specifically, the smallest accuracy has been observed for *CL7* (66%) and *CM7* (68%). In case of *CL7*, the misclassification results are 2% as *CL4* (same in-plane location, different interface of delamination), 12% as *CM7* (same interface, different in-plane location), 5% as *CM4* (different in-plane location, different interface), 2% as *CU7* (same interface, different in-plane location), 12% as *BL7* (same interface, different in-plane location), and 1% as *BM4* (different in-plane location, different interface). Herein, major misclassification is due to confusion between delaminations at the less severe interface along the thickness i.e., *D_7_*. Same is the case for misclassifications of *CM7*.

**Figure 8:**
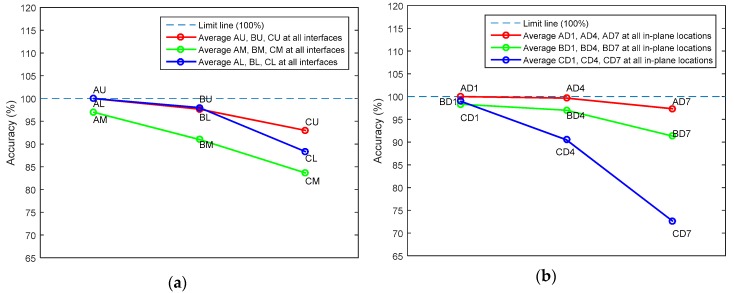
Average predictive performance of the pre-trained CNN with respect to: (**a**) in-plane location of delamination; (**b**) through-the-thickness interface of delamination.

The pre-trained CNN model classified the healthy and delaminated smart composite laminates with a classification accuracy of 94.5%. To get an idea of the classification performance regarding the in-plane location (*A*, *B*, *C*) and through-the-thickness interface (*D_1_*, *D_4_*, *D_7_*) of delamination, the classification accuracy is averaged according to the in-plane location and through-the-thickness interface as shown by Figure 8. 

For the in-plane location of delamination (Figure 8a), it is observed that the inner delaminations (*AM*, *BM*, *CM*) are difficult to detect compared with the edge delaminations (*AU*, *AL*, *BU*, *BL*, *CU*, *CL*). Owing to the boundary condition of inner and edge delaminations, the result is consistent with the physics of the problem. In addition, delaminations that occur near the free end are difficult to detect than the one that occurs near the fixed end. This might be due to the high strain near the fixed end or proximity of sensors to the delaminations near the fixed end or both [60]. From Figure 8b, the difficulty level of delamination detection increases as through-the-thickness interface of delamination is shifted from the mid-plane interface towards the free surfaces. This is because structural stiffness is more severely affected by delamination at the mid-plane than delamination of the same size away from the mid-plane interface. 

Contemporary research on delamination assessment (i.e., detection, quantification, and localization) in laminated composites focuses mainly on the use of high-frequency guided-waves (e.g., Lamb waves), acoustic emission/ultrasonic, and mode shape curvatures [61,62,63,64]. However, dealing with high-frequency signals may often cause difficulties in terms generation in a specific high-frequency band, high sampling rate, acquisition, storage, a requirement of the controlled experimental environment, and complex signal processing [41,65]. Similarly, acquiring mode shapes experimentally is an expensive and difficult task. On the contrary, low-frequency structural vibration is relatively easier to deal with due to its low sampling rate, small storage requirement, and ease of measurement through piezoelectric sensors. In addition, structural vibration is produced during operation of the component/structure and no input source of excitation is required. However, in most of the published literature, the parameters of structural vibration (e.g., natural frequency, modal damping) have been used for the global assessment (i.e., presence) of delamination [66,67,68,69]. The results of the methodology proposed in this work show the possibility to employ low-frequency structural vibration for the global (i.e., detection) and local assessment (i.e., localization, quantification) of delamination in smart composite laminates. In addition, the proposed methodology does not require labor-intensive hand-crafted features and automatically extract discriminative features for the assessment of delaminations in smart composite laminates. The key contribution of the current work is that delamination has been localized by assessing low-frequency structural vibration responses via a convolutional neural network (CNN). Although the large amount of damaged data for the CNN has been produced via numerical simulations, the proposed approach would be extended to real application with limited damaged data through data augmentation and transfer learning in future work. 

## 6. Conclusions

This paper proposed a deep learning framework for the assessment of delamination in smart composite laminates using low-frequency structural vibration responses. The initial assessment of transient responses revealed that multifarious excitations caused more variance in the response behavior than the variance caused by delamination in the structure. A convolutional neural network (CNN) was designed to automatically extract discriminative features from the vibration-based spectrograms and use those features to classify the healthy and delaminated smart composite laminates. The proposed architecture of the CNN showed a training accuracy of 99.9%, a validation accuracy of 97.1%, and a test accuracy of 94.5%. The test confusion chart of the pre-trained CNN revealed that the proposed approach not only distinguished the healthy and delaminated smart composite laminates with 100% accuracy, but also identified the more severe cases of delamination with 95%~100% accuracy. Furthermore, it was found that the detectability of delamination becomes difficult as the in-plane location of delamination is moved from the clamped end of the plate towards the free end and when through-the-thickness interfaces are moved from the mid-plane interface towards the free surfaces. Results of the proposed approach were found to be consistent with the physics of the problem. The proposed approach does not require the labor-intensive process of hand-crafted discriminative features and requires only low-frequency structural vibration response for the global and local assessment of delamination in smart composite laminates. In the future, the proposed approach would be extended to assess multiple damages (e.g., single and multiple delaminations, crack in matrix material, partial debonding of transducer) in cross-paly and angle-ply smart composite laminates. In addition, the proposed deep learning strategy could be used to bridge the gap between simulations, lab scale experiments, and large-scale structures of composite materials via transfer learning or data augmentation.

## Figures and Tables

**Table 1:** Material properties of a single lamina of the laminated plate.

E_1_	E_2_, E_3_	G_12_, G_13_	G_23_	ρ	ν_12_, ν_13_	ν_23_
372 GPa	4.12 GPa	3.99 GPa	3.6 GPa	1788.5 kg/m^3^	0.275	0.42

**Table 2:** Material properties of the piezoelectric sensors and actuator.

E	ν	ρ	d_31_, d_32_	d_24_, d_15_	d_36_
69 GPa	0.31	7700 kg/m^3^	179 × 10^−12^ C/N	−741 × 10^−12^ C/N	0

## Appendix A

(A1) $$\left[L_{u}\right]=\left[\begin{array}{cccccccc} 1 & 0 & C_{1}^{k}\frac{\partial }{\partial x}+D_{1}^{k}\frac{\partial }{\partial y} & A_{1}^{k} & B_{1}^{k} & H\left(z-z_{k}\right) & \bar{0} & E_{1}^{k}\frac{\partial }{\partial x}+F_{1}^{k}\frac{\partial }{\partial y}\\ 0 & 1 & C_{2}^{k}\frac{\partial }{\partial x}+D_{2}^{k}\frac{\partial }{\partial y} & A_{2}^{k} & B_{2}^{k} & \bar{0} & H\left(z-z_{k}\right) & E_{2}^{k}\frac{\partial }{\partial x}+F_{2}^{k}\frac{\partial }{\partial y}\\ 0 & 0 & 1 & 0 & 0 & \bar{0} & \bar{0} & H\left(z-z_{k}\right) \end{array}\right]$$

(A2) $$\left[L_{\varepsilon }\right]=\left[\begin{array}{cccccccc} \frac{\partial }{\partial x} & 0 & C_{1}^{k}\frac{\partial^{2}}{\partial x^{2}}+D_{1}^{k}\frac{\partial^{2}}{\partial x\partial y} & A_{1}^{k}\frac{\partial }{\partial x} & B_{1}^{k}\frac{\partial }{\partial x} & H\left(z-z_{j}\right)\frac{\partial }{\partial x} & \bar{0} & E_{1}^{k}\frac{\partial^{2}}{\partial x^{2}}+F_{1}^{k}\frac{\partial^{2}}{\partial x\partial y}\\ 0 & \frac{\partial }{\partial y} & C_{2}^{k}\frac{\partial^{2}}{\partial x\partial y}+D_{2}^{k}\frac{\partial^{2}}{\partial y^{2}} & A_{2}^{k}\frac{\partial }{\partial y} & B_{2}^{k}\frac{\partial }{\partial y} & \bar{0} & H\left(z-z_{j}\right)\frac{\partial }{\partial y} & E_{2}^{k}\frac{\partial^{2}}{\partial x\partial y}+F_{2}^{k}\frac{\partial^{2}}{\partial y^{2}}\\ 0 & 0 & C_{2,z}^{k}\frac{\partial }{\partial x}+\left(1+D_{2,z}^{k}\right)\frac{\partial }{\partial y} & A_{2,z}^{k} & B_{2,z}^{k} & \bar{0} & \bar{0} & E_{2,z}^{k}\frac{\partial }{\partial x}\left(F_{2,z}^{k}+H\left(z-z_{j}\right)\right)\frac{\partial }{\partial y}\\ 0 & 0 & \left(C_{1,z}^{k}+1\right)\frac{\partial }{\partial x}+D_{1,z}^{k}\frac{\partial }{\partial y} & A_{1,z}^{k} & B_{1,z}^{k} & \bar{0} & \bar{0} & \left[E_{1,z}^{k}+H\left(z-z_{j}\right)\right]\frac{\partial }{\partial x}+E_{1,z}^{k}\frac{\partial }{\partial y}\\ \frac{\partial }{\partial y} & \frac{\partial }{\partial x} & C_{2}^{k}\frac{\partial^{2}}{\partial x^{2}}+\left(C_{1}^{k}+D_{1}^{k}\right)\frac{\partial^{2}}{\partial x\partial y}+D_{1}^{k}\frac{\partial^{2}}{\partial y^{2}} & A_{1}^{k}\frac{\partial }{\partial y}+A_{2}^{k}\frac{\partial }{\partial x} & B_{1}^{k}\frac{\partial }{\partial y}+B_{2}^{k}\frac{\partial }{\partial x} & H\left(z-z_{j}\right)\frac{\partial }{\partial y} & H\left(z-z_{j}\right)\frac{\partial }{\partial x} & E_{2,z}^{k}\frac{\partial^{2}}{\partial x^{2}}+\left(E_{1}^{k}+F_{2}^{k}\right)\frac{\partial^{2}}{\partial x\partial y}+F_{1}^{k}\frac{\partial^{2}}{\partial y^{2}} \end{array}\right]$$

(A3) $$\left\{L_{\phi }^{p}\right\}=\left[\begin{array}{cc} 1-4\frac{{\left(z-z_{0}^{p}\right)}^{2}}{{\left(h^{p}\right)}^{2}} & -\left(z-z_{0}^{p}\right)+4\frac{{\left(z-z_{0}^{p}\right)}^{3}}{{\left(h^{p}\right)}^{2}} \end{array}\right]$$

(A4) $$\left[L_{E}^{p}\right]=\left[\begin{array}{cc} \left(1-4\frac{{\left(1-z_{0}^{p}\right)}^{2}}{{\left(h^{p}\right)}^{2}}\right)\frac{\partial }{\partial x} & \left(4\frac{{\left(1-z_{0}^{p}\right)}^{3}}{{\left(h^{p}\right)}^{2}}-\left(z-z_{0}^{p}\right)\right)\frac{\partial }{\partial x}\\ \left(1-4\frac{{\left(1-z_{0}^{p}\right)}^{2}}{{\left(h^{p}\right)}^{2}}\right)\frac{\partial }{\partial y} & \left(4\frac{{\left(1-z_{0}^{p}\right)}^{3}}{{\left(h^{p}\right)}^{2}}-\left(z-z_{0}^{p}\right)\right)\frac{\partial }{\partial y}\\ -8\frac{\left(1-z_{0}^{p}\right)}{{\left(h^{p}\right)}^{2}} & 12\frac{{\left(1-z_{0}^{p}\right)}^{2}}{{\left(h^{p}\right)}^{2}}-1 \end{array}\right]$$

(A5) $$V_{b}=4{\bar{\phi }}^{p}\frac{{\left(1-z_{0}^{p}\right)}^{3}}{{\left(h^{p}\right)}^{3}}$$

(A6) $$\left\{F_{b}\right\}={\left[\begin{array}{ccc} 0 & 0 & 12{\bar{\phi }}^{p}\frac{{\left(1-z_{0}^{p}\right)}^{2}}{{\left(h^{p}\right)}^{3}} \end{array}\right]}^{T}$$
